# Study on the Road Performance and Compaction Characteristics of Fiber-Reinforced High-RAP Plant-Mixed Hot Recycled Asphalt Mixtures

**DOI:** 10.3390/polym16142016

**Published:** 2024-07-15

**Authors:** Chunfeng Zhu, Yongyong Yang, Kai Zhang, Di Yu

**Affiliations:** 1 College of Civil Engineering, Jilin Jianzhu University, Changchun 130118, China; zhuchunfeng@jlju.edu.cn (C.Z.);; 2 Jiangxi Transportation Institute Co., Ltd., Nanchang 330200, China; 3 School of Civil Engineering and Architecture, East China Jiaotong University, Nanchang 330013, China

**Keywords:** recycled asphalt mixture, basalt fiber, polyester fiber, road performance, compaction characteristics

## Abstract

Recycled asphalt pavement (RAP) mixtures are widely adopted due to their significant economic and social benefits from utilizing pavement recycling materials. This study incorporates basalt fibers (BF) and polyester fibers (PF) into plant-mixed hot recycled asphalt mixtures to analyze their enhancement effects on the high-temperature, low-temperature, and fatigue performance at different RAP content levels. Additionally, the study investigates the impact of fiber and RAP additions on the compaction characteristics of the mixtures using gyratory compaction tests, aiming to increase the RAP content of plant-mixed hot recycled asphalt mixtures. Experimental results demonstrate that at 30% and 50% RAP content levels, basalt fibers exhibit more pronounced enhancement effects on the performance of recycled asphalt mixtures compared to polyester fibers. Incorporating basalt fibers increases the fracture energy of recycled asphalt mixtures by 8.63% and 13.9%, and improves fatigue life by 154% and 135%, respectively. Moreover, the addition of both types of fibers increases compaction difficulty, with polyester fibers showing a more significant influence on the compaction energy index (CEI).

## 1. Introduction

In recent years, with the rapid development of road infrastructure, the utilization of RAP has become a significant approach to addressing resource wastage and environmental pollution. Plant-mixed hot recycled asphalt mixtures have attracted widespread attention due to their ability to significantly conserve resources and reduce construction costs. However, the application of high RAP content often leads to a decline in the performance of asphalt mixtures. To tackle this issue, fiber reinforcement technology has been applied to recycled asphalt mixtures. Polyester fibers and basalt fibers, known for their excellent mechanical properties and chemical stability, have become crucial additives in modified asphalt mixtures, particularly in styrene-butadiene-styrene (SBS) polymer-modified asphalt mixtures. The addition of fibers is expected to further enhance the overall performance of recycled asphalt mixtures [1,2,3].

High RAP content asphalt mixtures are prone to increased fatigue and low-temperature cracking during their service life [4]. Zhou et al.’s study indicates that an increase in RAP content enhances the fatigue accumulation rate of asphalt mixtures, thereby reducing their fatigue life [5]. Shu et al. evaluated the fatigue characteristics of RAP mixtures using the superpave indirect tension test and the dissipated creep strain energy threshold. Their study found that an increase in RAP content lowers the dissipated creep strain energy threshold value, indicating that the energy required for asphalt mixture cracking decreases with higher RAP content. This suggests that increasing RAP content damages the fatigue life of recycled asphalt mixtures [6]. Furthermore, in cold regions, increasing RAP content may reduce the low-temperature performance of asphalt mixtures. The aged asphalt in RAP increases the stiffness and brittleness of the asphalt mixture, reducing the stress relaxation capacity of asphalt pavements, and thereby increasing the risk of low-temperature cracking [7,8]. Zhang et al. evaluated the low-temperature characteristics of RAP mixtures using the Direct Tensile Stress Relaxation Test. Their study indicated that under constant strain loading conditions, as the RAP content increases, the relaxation time of the asphalt mixture becomes longer, resulting in poorer low-temperature relaxation performance. Moreover, at a 50% RAP content, the relaxation performance of the asphalt mixture was severely compromised [9]. Furthermore, the increase in RAP content reduces the moisture damage resistance of recycled asphalt mixtures. Zeng et al. employed the standard contabro test and dynamic modulus test on moisture-induced sensitivity test-treated samples to assess the water stability after dynamic water scouring. The results demonstrated that as the RAP content increased, the water stability of the recycled asphalt mixtures declined [10].

On the one hand, adding rejuvenators to recycled asphalt mixtures can effectively reactivate aged asphalt, improve the blending between virgin and RAP binders, and enhance the overall performance of recycled asphalt mixtures [11,12]. On the other hand, for asphalt mixtures with high RAP content, the addition of fibers can further enhance the pavement performance of recycled asphalt mixtures. Fibers, as effective modifiers, form a stable spatial network structure with asphalt mixtures due to their excellent tensile strength. This enhances the toughness and crack resistance of asphalt mixtures [3,13]. Most fibers used in asphalt mixtures can be broadly classified as either inorganic (such as basalt fibers and glass fibers) or organic (such as polypropylene fibers and polyester fibers) [14,15,16]. Current research indicates that the addition of fibers in asphalt can effectively improve its rheological properties, and increase its softening point and viscosity, thereby enhancing resistance to rutting [17,18]. Moreover, dry-mixing fibers with asphalt mixtures can also improve the fatigue and low-temperature performance of asphalt mixtures [19]. Zhu and Yu analyzed the impact of basalt fibers and diatomite-modified asphalt mixtures, showing that the fatigue life and low-temperature crack resistance of basalt fiber/diatomite composite-modified asphalt mixtures were significantly improved [20,21]. Park et al. evaluated fracture energy and toughness as indicators using indirect tensile tests. The results showed that an appropriate amount of steel fibers can significantly enhance the low-temperature crack resistance of asphalt concrete [22]. Li et al. conducted experiments to evaluate various fiber types for enhancing asphalt mixture performance. Their findings indicate that the addition of basalt fibers to RAP mixtures notably enhances high-temperature stability and fatigue resistance. Polyester fibers demonstrated considerable enhancement in moisture damage resistance, while lignin fibers exhibited notable improvements in low-temperature performance [23]. Similarly, Wu et al. evaluated the performance of basalt fibers in asphalt mixtures using beam bending tests. The results indicate that basalt fibers compensate for the hardening effect of RAP caused by low temperatures, thereby enhancing the crack resistance of RAP mixtures in cold environments [13]. Yu et al. demonstrated that incorporating 0.25% of the mass of polyester fibers into asphalt mixtures containing 50% RAP improves their fatigue and low-temperature performance [24]. Ziari et al. investigated the crack resistance of glass fiber-reinforced recycled asphalt mixtures, using the J integral (Jc) and fracture toughness (KIC) from the SCB test as evaluation metrics. The results indicated that in low-temperature environments, increasing the RAP content and adding glass fibers can improve fracture performance [15].

Generally, compaction of asphalt mixtures refers to the process of transforming loose particle states into a viscoelastic state. Poor compaction of RAP mixtures may lead to severe rutting, poor fatigue resistance, and premature moisture damage [25,26,27,28,29]. Based on current research, the addition of RAP makes asphalt mixtures more difficult to compact. Ma et al. used the superpave gyratory compactor to calculate the compactability based on the compaction energy ratio. Their findings indicate that increasing RAP content at the same as heating the temperature results in increased difficulty in compaction. This effect is particularly pronounced in mixtures containing SBS-modified asphalt compared to unmodified asphalt mixtures [30]. Singh Dharamveer et al. pointed out that the addition of RAP increases the mixing and compaction temperatures during the construction phase. Therefore, additional warm mix additives are needed to lower the production temperature and improve compaction [31]. However, during the compaction process, the presence of fibers affects the flowability and uniformity of RAP mixtures, thereby further increasing the difficulty of compaction. Giustozzi et al. indicated that a high fiber content can increase the compaction difficulty of RAP mixtures, leading to excessive air void content [32].

This study prepared control asphalt mixtures with 0%, 30%, and 50% RAP content, as well as basalt fiber-modified asphalt mixtures and polyester fiber-modified asphalt mixtures with 30% and 50% RAP content, respectively. Various performance tests, including rutting, dynamic water scouring, indirect tensile fatigue, semi-circular bending, and gyratory compaction tests, were conducted to investigate the effects of polyester fibers and basalt fibers on the performance of asphalt mixtures with high RAP content. Additionally, the study analyzed how the addition of these fibers and RAP affects the compaction difficulty of asphalt mixtures, aiming to provide design references for increasing the allowable RAP content in asphalt mixtures.

## 2. Materials and Experimental Methods

### 2.1. Materials

#### 2.1.1. Fiber

**Figure 1:**
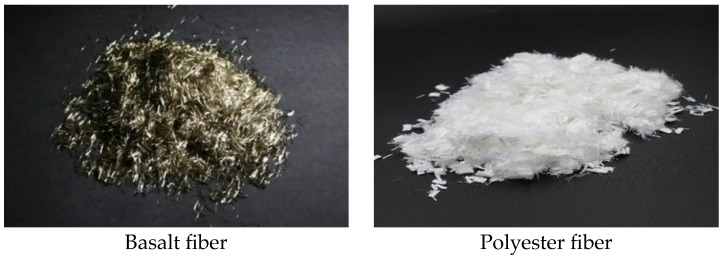
Fiber sample.

The types of fibers used in this study include basalt fibers and polyester fibers. The basalt fibers were procured from China Jilin Tongxin Basalt Technology Co., Ltd., while the polyester fibers were sourced from China Yancheng Oulu Hua Fiber Technology Co., Ltd. The shapes and appearances of these two fibers are depicted in Figure 1, and their respective technical specifications are detailed in Table 1 and Table 2.

#### 2.1.2. Virgin Binder

The virgin asphalt is SBS-modified asphalt (I-D), and its performance was evaluated according to specifications in [33]. The performance characteristics of this asphalt are detailed in Table 3, and all technical specifications meet the requirements of the specification [34].

#### 2.1.3. RAP and Aggregates

This study used refined crushed and screened RAP materials provided by a certain expressway in Jiangxi Province. Two gradations, (0–5 mm) and (5–11 mm), were selected as the RAP aggregates. According to the specification [33], the RAP was extracted using the extraction method to obtain reclaimed aggregate and aged asphalt. The properties of the aged asphalt are shown in Table 4, and the reclaimed aggregate gradations are presented in Table 5. Limestone aggregate was used as the new aggregate, divided into #1 (0–3 mm), #2 (3–6 mm), #3 (6–11 mm), and #4 (11–16 mm). Specific indicators are detailed in Table 6.

#### 2.1.4. Rejuvenator

In this study, the commercial rejuvenator Evoflex8182 was used, and its technical specifications are provided in Table 7. Based on the performance indicators of aged asphalt, the amount of rejuvenator is determined to be 6% of the aged asphalt content.

#### 2.1.5. Gradation Design

**Figure 2:**
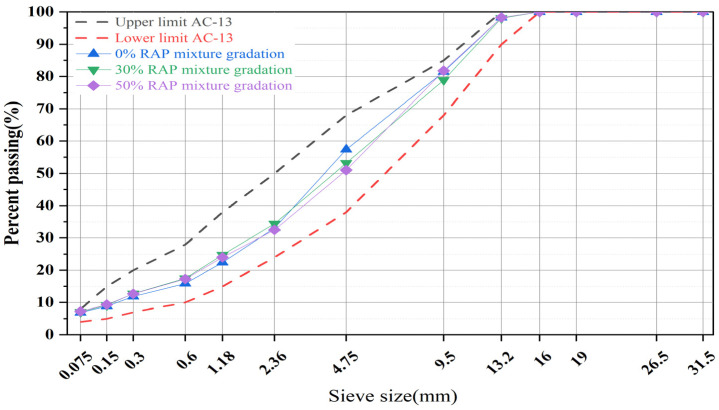
Gradation curve.

**Figure 3:**
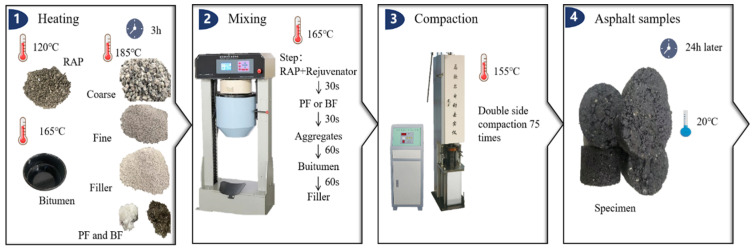
Preparation of the recycled asphalt mixture.

This study employed the Marshall design method to prepare asphalt mixture specimens, with all mixtures designed according to the grading range recommended by [34]. The asphalt mixture gradation is shown in Figure 2, and the molding method is illustrated in Figure 3. The optimal new asphalt contents for asphalt mixtures with RAP contents of 0%, 30%, and 50% were determined to be 4.6%, 3.5%, and 2.9%, respectively. Furthermore, to ascertain the impact of fibers on asphalt mixtures, the optimal new asphalt contents were controlled at 3.5% and 2.9% when fibers were added to asphalt mixtures containing 30% and 50% RAP, respectively. Furthermore, based on existing research conclusions, fiber content generally ranges from 0.1% to 0.6% of the total mass of the asphalt mixture [35,36]. To compare the effects of two different types of fibers on RAP material performance, a fiber content of 0.3% of the total mass of the asphalt mixture was selected.

### 2.2. Experiments and Methods

#### 2.2.1. High-Temperature Rutting Test

High-temperature stability refers to the ability of asphalt mixtures to resist shear and deformation at high temperatures. In this study, the dynamic stability (DS) from rutting tests was used as an indicator to evaluate the high-temperature stability of the asphalt mixtures. The rutting tests were conducted according to the Chinese standard [33]. The test used slab specimens with dimensions of 300 mm × 300 mm × 50 mm. The wheel speed was set to 42 cycles per minute, with a default pressure of 0.7 MPa, and the test temperature was 60 °C. The specimens were kept warm at the set temperature for more than 5 h, but not exceeding 12 h. Generally, higher DS values indicate superior rutting performance.

The dynamic stability can be calculated by Equation (1):

(1) $$DS=\frac{\left(t_{2}-t_{1}\right)\times N}{d_{2}-d_{1}}\times C_{1}\times C_{2}$$

where d_1_ is the rut depth (mm) at 45 min; d_2_ is the rut depth (mm) at 60 min; t_1_ is the 45th min; t_2_ is the 60th min; $C_{1}$ and $C_{2}$ are experimental coefficients, and in this study, $C_{1}$ = $C_{2}$ = 1.0; and N is the number of wheel passes per minute, N = 42 passes/min.

#### 2.2.2. Dynamic Water Scouring Test

**Figure 4:**
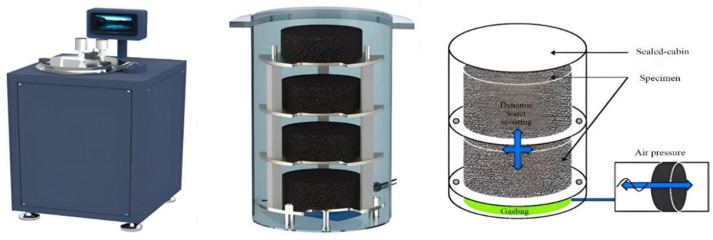
Moisture-induced sensitivity tester.

Dynamic pore water pressure-induced asphalt pavement moisture damage is more common in areas with abundant rainfall. When vehicles travel on roads with surface runoff, it creates a dynamic pore water pressure environment, leading to erosion processes such as dynamic water scouring on the asphalt pavement [37]. Therefore, this study conducted dynamic water scouring tests to simulate the effects of vehicle loading-induced dynamic water erosion on asphalt pavement under the conditions of hot and rainy summers. The moisture-induced sensitivity tester equipment shown in Figure 4 was used in this study. The dynamic water pressure adjustment cycle was approximately 4 s, with a constant loading frequency of 0.25 Hz. The test was set to 3500 cycles, with a pressure value of 276 kPa, and a temperature setting of 60 °C. After the test, the specimens were placed in a constant temperature water bath at 25 °C ± 1 °C for 2 h. The indirect tensile strength test was used to evaluate the change in strength before and after dynamic water scouring.

The indirect tensile strength can be calculated by Equations (2) and (3):

(2) $$S_{T1}=0.006287P_{T1}/h_{1}$$

(3) $$S_{T2}=0.006287P_{T2}/h_{2}$$

where $S_{T1}$ is the indirect tensile strength of the first group of specimens before dynamic water scouring (MPa); $S_{T2}$ is the indirect tensile strength of the second group of specimens after dynamic water scouring (MPa); $P_{T}$ is the maximum load of a single specimen in the test (N); and h is the specimen height (mm).

The indirect tensile strength ratio can be calculated by Equation (4):

(4) $$ITSR=\frac{\bar{S_{T1}}}{\bar{S_{T2}}}\times 100\%\bar{S_{T2}}$$

where ITSR is the indirect tensile strength ratio (%); $\bar{S_{T1}}$ is the average value of the first group of specimens before dynamic water scouring (MPa); and $\bar{S_{T2}}$ is the average value of the second group of specimens after dynamic water scouring (MPa).

#### 2.2.3. Semi-Circular Bending Test

**Figure 5:**
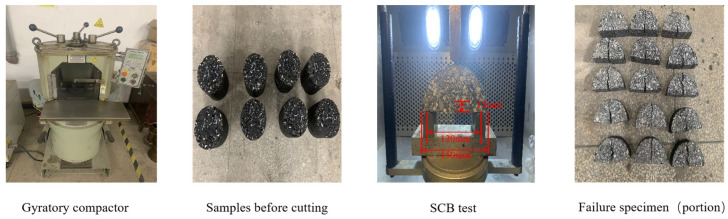
SCB test process.

The inclusion of a high proportion of RAP increases the stiffness and brittleness of asphalt mixtures, making them more susceptible to brittle failure in low-temperature environments. This study utilized the semi-circular bend (SCB) test to assess the low-temperature cracking resistance of asphalt mixtures. Cylindrical asphalt mixture specimens with a diameter of 150 mm and a height of 160 mm were prepared using a gyratory compactor. These specimens were subsequently cut into semi-circular specimens with a diameter of 150 mm, a radius of 75 mm, a thickness of 50 mm, and pre-cut notches with a depth of 15 mm and a width of 1.5 mm. Each cylindrical specimen could be cut into four semi-circular specimens. The temperature was set at −10 °C, and the specimens were kept in a temperature-controlled environment for at least 6 h. A loading rate of 1.2 mm/min was applied, and the test was terminated when the load was less than 0.1 kN, with continuous recording of the load–displacement curve. In this study, fracture energy (G_f_) and fracture toughness were computed to characterize the cracking behavior of fiber-reinforced asphalt mixtures. The experimental procedure is illustrated in Figure 5.

The fracture work can be calculated by Equation (5):

(5) $$W_{f}=\int Pdu$$

where P is the applied load (kN) and u is the displacement of load line (mm).

The fracture energy can be calculated by Equation (6):

(6) $$G_{f}=\frac{W_{f}}{Area_{lig}}$$

where $G_{f}$ is the fracture energy (J/m^2^); $W_{f}$ is the fracture work (J); $Area_{lig}$ is the specimen ligament area (mm^2^) $Area_{lig}$ = (r−a)×t; r is the specimen radius (mm); a is the crack depth (mm); and t is the specimen thickness (mm).

The fracture toughness can be calculated by Equation (7):

(7) $$K_{IC}=\frac{P}{2rt}Y_{I}\sqrt{\pi a}$$

where $K_{IC}$ is the fracture toughness (MPa × m^1/2^) and $Y_{I}$ is the standard stress intensity factor.

#### 2.2.4. Indirect Tensile Fatigue Test

**Figure 6:**
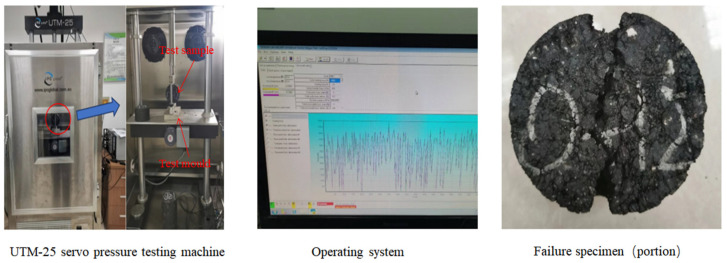
Indirect tensile fatigue test process.

This study utilized the indirect tensile fatigue test to assess the fatigue performance of fiber-reinforced asphalt mixtures. The indirect tensile fatigue test was conducted using a UTM-25 servo-hydraulic testing machine by the Australian manufacturer IPC Global. Specimens were prepared by gyratory compaction with 100 gyrations, resulting in specimens with a diameter of 101.6 mm and a thickness of 63.5 mm. The tests were performed at a temperature of 25 °C in stress control mode, applying a pulsating semi-sine wave load at a frequency of 10 Hz with an interval time of 0.1 s, and a stress ratio of 0.4. The experimental process is depicted in Figure 6 [38].

#### 2.2.5. Gyratory Compaction Test

Compaction density is a critical indicator during the construction of asphalt mixtures and plays a crucial role in the pavement’s longevity. Asphalt pavements with poor compaction density are more prone to damages such as cracking, rutting, and stripping [39]. Additionally, the inclusion of RAP and fibers also affects the compaction difficulty of asphalt mixtures. This study conducted gyratory compaction tests following the guidelines outlined in Chinese standards [33]. The evaluation of the compaction characteristics of asphalt mixtures concerning the influence of fibers and RAP utilized indicators such as the CEI, K value, and air void content [40]. The CEI refers to the energy required to compact the asphalt mixture from a loose state to a preliminary compaction (92%). A higher CEI signifies increased compaction difficulty for the asphalt mixtures. Analysis from gyratory compactor data reveals that the compaction density of asphalt mixtures follows a semi-logarithmic curve across compaction cycles. The average slope of this curve, represented by the K value, indicates the compaction rate: a higher K value suggests better compaction performance. In this experiment, the initial compaction cycle was set to 8 cycles, and the design compaction cycle was 100 cycles. To ensure that the air void content of the specimens did not vary due to changes in specimen mass, the mass of all specimens was standardized to approximately 1180 g.

The CEI can be calculated by Equation (8):

(8) $$CEI=\sum_{n_{0}}^{n_{i}=N_{G=92\%}}\left(G_{ni}-G_{0}\right)d\left(n_{i}\right)$$

where $N_{G=92\%}$ is the number of compaction cycles corresponding to 92% compaction density; and $G_{ni}$ is the compaction density curve function.

The K value can be calculated by Equation (9):

(9) $$K=\frac{G_{100}-G_{8}}{Ln100-Ln8}$$

where $G_{i}$ is the density of the ith gyratory compaction.

## 3. Discussion of Test Results

### 3.1. Results of High-Temperature Rutting Test

**Figure 7:**
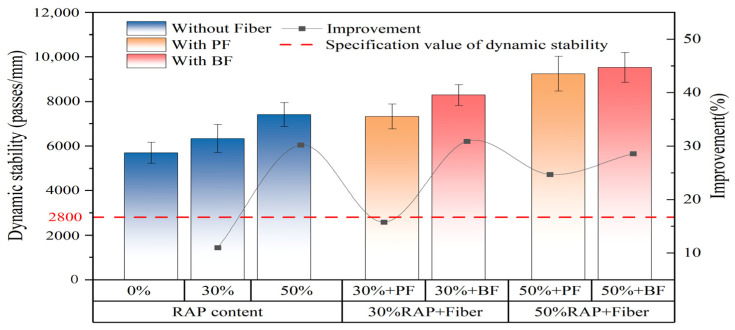
High-temperature rutting test results.

Figure 7 illustrates the improvement in the dynamic stability of asphalt mixtures with different RAP contents by basalt fiber and polyester fiber. From the figure, it can be seen that in the control group without fibers, the increase in RAP content also improves the dynamic stability of the asphalt mixture. This may be due to the increase in aged asphalt content in the mixture, which increases stiffness, manifesting as enhanced hardness and improved resistance to high-temperature shear [41]. Additionally, as indicated by the black lines in the graph, polyester fibers improved the dynamic stability by 15.7% and 24.7% for the 30% and 50% RAP mixtures without fiber, respectively. This suggests that increasing RAP content did not adversely affect the enhancement effect of polyester fibers. In contrast, basalt fibers showed a diminishing enhancement effect on asphalt mixture dynamic stability with increasing RAP content, improving dynamic stability by 30.1% and 28.6%, respectively. However, overall, basalt fibers still significantly enhanced the dynamic stability of recycled asphalt mixtures at high RAP content. This effect may be attributed to the extremely smooth surfaces of polyester fibers, which result in poor asphalt adsorption. In contrast, basalt fibers exhibit a certain degree of surface roughness at the microscopic level and demonstrate good dispersibility within asphalt mastics. These characteristics enhance asphalt adsorption, thereby improving the viscosity and high-temperature stability of the asphalt mastics [42]. Additionally, in asphalt mixtures with high RAP content, the increased aged asphalt reduces the amount of asphalt that fibers can easily adsorb, thereby reducing the reinforcement effect of fibers [3].

### 3.2. Results of Dynamic Water Scouring Test

**Figure 8:**
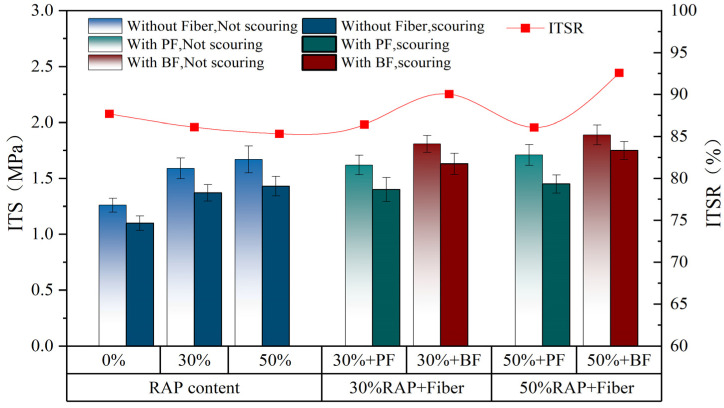
Dynamic water scouring results.

Figure 8 presents the ITS and ITSR before and after dynamic water scouring. As indicated by the red line in Figure 8, in the control group without fibers, there is a noticeable decrease in ITSR of the asphalt mixture with increasing RAP content. When the RAP content increases from 0% to 50%, the ITSR value decreases from 87.7% to 85.3%. However, after the addition of polyester fiber and basalt fiber, ITSR shows an overall increasing trend, which is consistent with the findings of Li et al. [23]. Fibers can enhance the bonding between asphalt and aggregate, improving the water damage resistance of asphalt mixtures. Notably, basalt fiber demonstrates the most significant improvement, increasing to 90.06% and 92.59% at RAP contents of 30% and 50%, respectively. In contrast, the enhancement effect of polyester fiber is relatively limited, with ITSR increasing to 86.42% and 86.1% at the same RAP contents. Additionally, according to the bar chart in Figure 8, both fibers enhance the ITS of specimens, with basalt fiber showing more pronounced improvement.

### 3.3. Results of Indirect Tensile Fatigue Test

**Figure 9:**
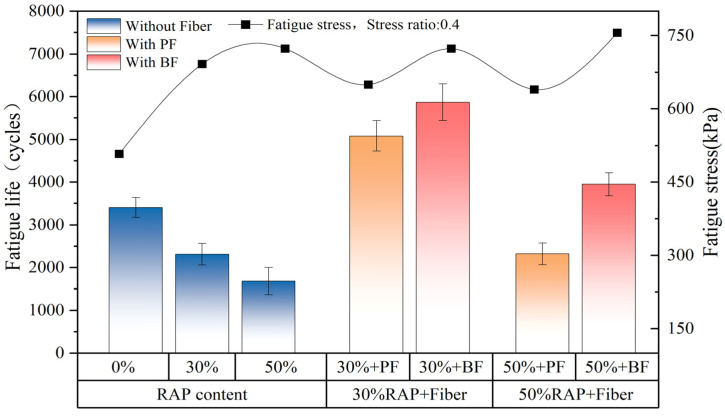
Indirect tensile fatigue results.

Figure 9 illustrates the improvement in fatigue life of asphalt mixtures with different RAP contents due to basalt fiber and polyester fiber, as well as the fatigue stress at a stress ratio of 0.4. As shown by the black line in Figure 9, in the control group without fibers, the tensile strength of the asphalt mixture increases with the rise in RAP content. This may be attributed to the higher RAP content, which leads to an increased presence of aged asphalt in the mixture, thereby increasing its hardness and brittleness. However, the enhancement effect of polyester fiber on the tensile strength of the asphalt mixture is not significant. As indicated by the bar chart in Figure 9, the addition of both fibers increases the fatigue life of the asphalt mixture. Basalt fiber improves the fatigue life by 154% and 135% at RAP contents of 30% and 50%, respectively. In comparison, polyester fiber increases the fatigue life by 119% and 38% at the same RAP contents. This is consistent with the findings of Zhu et al. [23], who reported that under the same stress ratios, asphalt mixtures containing basalt fibers have higher fatigue life than those containing polyester fibers. One reason for this may be that, similar to high-temperature rutting tests, basalt fibers can better adsorb asphalt, forming a stable spatial network structure. Another reason may be that basalt fibers have higher tensile strength and modulus of elasticity compared to polyester fibers, thereby significantly enhancing the fatigue life of the asphalt mixture.

### 3.4. Results of Semi-Circular Bending Test

**Figure 10:**
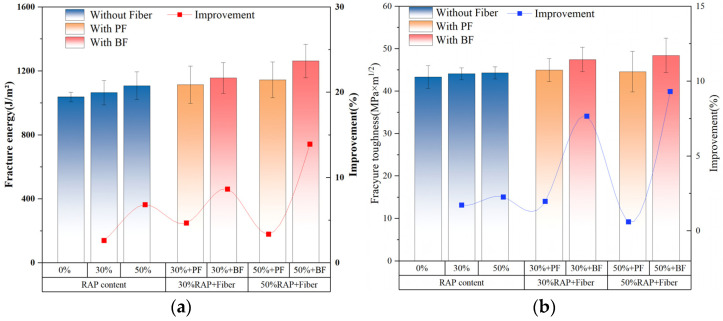
SCB test results: (**a**) fracture energy, (**b**) fracture toughness.

Figure 10 presents the results of semi-circular bending tests on asphalt mixtures with basalt fiber and polyester fiber at different RAP contents. At −10 °C, the results of fracture energy testing are shown in the bar chart in Figure 10a. In the control group without fibers, the fracture energy of the asphalt mixture increases with the rise in RAP content. This phenomenon occurs because, at −10 °C, the asphalt mixture exhibits a brittle state with a high modulus, and the displacement at failure is minimal. Consequently, the material is more influenced by stress than by strain [15,43]. Increasing RAP content under low-temperature conditions increases the hardness and load-bearing capacity of the asphalt mixture, thereby enhancing fracture energy. Additionally, as indicated by the red line in Figure 10a, the addition of both types of fibers can increase the fracture energy of the asphalt mixture. Compared to polyester fiber, basalt fiber shows a more pronounced enhancement effect. In contrast, basalt fibers increased the fracture energy of asphalt mixtures with 30% and 50% RAP content by 8.63% and 13.9%, respectively, compared to those without fiber reinforcement. Meanwhile, polyester fibers at the same RAP content enhanced the fracture energy of asphalt mixtures by 4.66% and 3.35%, respectively. This enhancement is attributed to the significant influence of interfacial strength on the fracture energy of asphalt mixtures. Basalt fibers, possessing higher surface energy, enable tighter bonding with the asphalt binder, thus achieving higher interfacial strength. Therefore, basalt fibers are more effective in enhancing the fracture energy of asphalt mixtures [44,45]. Similarly, comparable results are observed in Figure 10b for fracture toughness. Fracture toughness is mainly influenced by the ultimate load, and the addition of RAP can improve the hardness of the mixture, thereby increasing the ultimate load. Moreover, basalt fiber may form a more stable network structure and have higher tensile strength in the mixture than polyester fiber, thus significantly enhancing the fracture toughness of the asphalt mixture under low-temperature conditions [42,46].

### 3.5. Results of Gyratory Compaction Test

**Figure 11:**
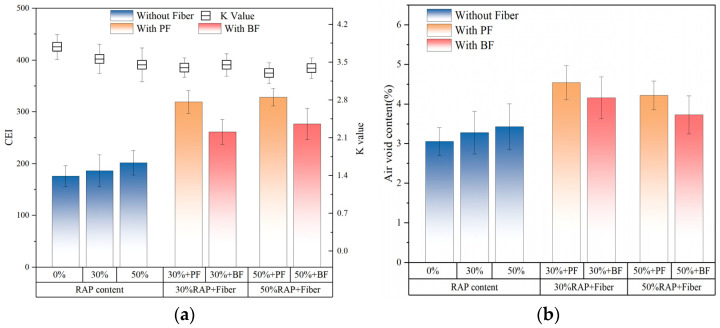
Compaction energy test results: (**a**) K value and CEI; (**b**) air void content.

As shown in Figure 11a, the increase in RAP content in the control group without fibers leads to an increase in aged asphalt in the asphalt mixture, resulting in higher asphalt viscosity and increased difficulty in sliding between aggregates. Consequently, the air void content of the asphalt mixture increases at the same compaction times. Additionally, both types of fibers also increase the air void content of the asphalt mixture. However, polyester fiber has a more significant impact on increasing the air void content compared to basalt fiber. As depicted in Figure 11b, The addition of RAP and fibers has reduced the compaction rate (K value) of the asphalt mixture. Furthermore, the addition of fibers and RAP increases the required CEI. Under the same RAP content, polyester fibers require a higher CEI. This may be due to the softer and more flexible texture of polyester fibers compared to basalt fibers, making polyester fibers less prone to sliding with the asphalt mixture during compaction, thereby generating greater resistance. This in turn reduces the compaction rate of the asphalt mixture and increases the energy required during compaction.

## 4. Conclusions

This study evaluates the effects of basalt fiber and polyester fiber on asphalt mixtures with high RAP content in terms of high-temperature stability, water stability, fatigue resistance, low-temperature crack resistance, and asphalt mixture compaction characteristics through indoor experiments. Based on the preceding analysis, the following conclusions can be drawn:

(1) As the RAP content increases, the dynamic stability of asphalt mixtures improves. Furthermore, the addition of polyester fiber and basalt fiber creates a spatial network structure within the asphalt mixture, enhancing its shear strength and improving its high-temperature stability. Basalt fiber demonstrates a more significant enhancement effect compared to polyester fiber;
(2) While the inclusion of RAP enhances the indirect tensile strength of asphalt mixtures before dynamic water immersion, the ITSR curve of asphalt mixtures indicates a decrease in water damage resistance with high RAP content. The incorporation of fibers can improve the water damage resistance of asphalt mixtures, with basalt fiber significantly enhancing this property compared to polyester fiber;
(3) An increase in RAP content notably decreases the fatigue performance of asphalt mixtures at a stress ratio of 0.4. The addition of polyester fiber or basalt fiber can effectively enhance the fatigue resistance of asphalt mixtures, with basalt fiber demonstrating a more significant improvement;
(4) At −10 °C, the inclusion of both types of fibers improves the crack resistance of asphalt mixtures. Basalt fiber exhibits a more substantial enhancement in fracture energy and fracture toughness at high RAP contents of 30% and 50%, respectively, compared to polyester fiber;
(5) According to indoor experiments simulating compaction processes, the incorporation of RAP and fibers increases the air void content of asphalt mixtures and decreases the compaction rate, thereby increasing the CEI. However, basalt fiber has a smaller impact on reducing the compaction rate and increasing the air void content and CEI compared to polyester fiber.

The primary findings of this study demonstrate that a high RAP content reduces the water damage resistance and crack resistance of asphalt mixtures. Incorporating fibers into asphalt mixtures effectively mitigates the performance decline caused by RAP. Among these fibers, basalt fiber exhibits the most significant improvement in the performance of asphalt mixtures with high RAP content compared to polyester fiber. However, the addition of fibers also increases the compaction difficulty of asphalt mixtures. Therefore, to enhance the performance of recycled pavement using fibers, it is advisable to increase the compaction temperature and number of compaction cycles of the asphalt mixture.

## Figures and Tables

**Table 1:** Basic technical indicators of basalt fiber.

Test Items	Unit	Test Results	Technical Requirements
Appearance	%	98.6	≥90
Fiber length	mm	6.03	6 ± 10%
Linear density	tex	225.1	225.8 ± 8%
Fiber diameter	mm	17.01	17 ± 10%
Combustible	——	Non-flammable in open flame	Non-flammable in open flame
Density	g/cm^3^	2.66	2.60–2.80
Modulus of elasticity	MPa	81.5 × 10^3^	≥7.5 × 10^3^
Elongation at break	%	2.4	2.4–3.1
Fracture strength	MPa	1675	1200–2200
Moisture content	%	0.036	≤0.2

**Table 2:** Basic technical indicators of polyester fiber.

Test Items	Unit	Test Results	Technical Requirements
Fiber diameter	μm	21.5	10–25
Fiber length	mm	6.0	6 ± 1.5
Tensile strength	MPa	518	≥500
Density	g/cm^3^	1.36	1.36 ± 0.04
Elongation at break	%	18.2	≥15
Heat resistance	——	220 °C, no volume change after 2 h	220 °C, no volume change after 2 h

**Table 3:** SBS-modified asphalt technical specifications.

Test Items	Unit	Standard	Test Value	Specification Value
Penetration	0.1 mm (@25 °C, 100 g, 5 s)	T0604	58.5	30–60
Ductility	Cm (@5 °C, 5 cm/min)	T0605	31.2	≥20
Softening point	°C	T0606	78.8	≥60
Viscosity at 135 °C	Pa·s	T0625	2.93	≤3

**Table 4:** Aged asphalt technical specifications.

Test Items	Unit	Standard	Test Value
Penetration	0.1 mm (@25 °C, 100 g, 5 s)	T0604	31.2
Ductility	Cm (@5 °C, 5 cm/min)	T0605	1.9
Softening point	°C	T0606	74.8
Viscosity at 135 °C	Pa·s	T0625	1.74

**Table 5:** The RAP aggregate gradation.

Sieve Size (mm)	16	13.2	9.5	4.75	2.36	1.18	0.6	0.3	0.15	0.075
	Gradation Passing Rate of Reclaimed Aggregates (%)									
0–5	100	100	100	96.22	78.52	62.22	46.06	32.78	24.38	18.44
5–11	100	100	99.13	28.68	12.34	10.49	8.60	6.90	5.61	3.50

**Table 6:** Original aggregate technical specifications.

Test Items	Unit	Test Value				Standard
		0–3 mm	3–6 mm	6–11 mm	11–16 mm	
Apparent relative density	g/cm^3^	2.613	2.709	2.708	2.705	≥2.60
Bulk relative density	g/cm^3^	2.613	2.631	2.658	2.688	——
Water absorption rate	%	0.75	0.54	0.44	0.31	——

**Table 7:** Technical index of the Evoflex8182 rejuvenator.

Test Items	Unit	Test Value	Standard
Viscosity at 60 °C	cst	9532	T0619
Flash point	°C	240.4	T0633
Saturates content	%	21.5	T0618
Aromatic content	%	41.5	T0618
Viscosity ratio before thin film oven test	——	1.3	T0619
Mass change before and after thin film oven test	%	0.8	T0609

## Data Availability

The testing and analysis data used to support the findings of this study are included within the article.
